# NOD2 Responds to Dengue Virus Type 2 Infection in Macrophage-like Cells Interacting with MAVS Adaptor and Affecting IFN-α Production and Virus Titers

**DOI:** 10.3390/pathogens13040306

**Published:** 2024-04-10

**Authors:** Diana Alhelí Domínguez-Martínez, Mayra Silvia Pérez-Flores, Daniel Núñez-Avellaneda, Jesús M. Torres-Flores, Gloria León-Avila, Blanca Estela García-Pérez, Ma Isabel Salazar

**Affiliations:** 1 Laboratorio de Inmunología Celular e Inmunopatogénesis, Departamento de Inmunología, Escuela Nacional de Ciencias Biológicas, Instituto Politécnico Nacional, Ciudad de México CP 11340, Mexico; alheli43@hotmail.com; 2 Unidad de Investigación en Virología y Cáncer, Hospital Infantil de México Federico Gómez, Ciudad de México CP 06720, Mexico; 3 Laboratorio Nacional de Vacunología y Virus Tropicales (LNVyVT), Escuela Nacional de Ciencias Biológicas Instituto Politécnico Nacional, Ciudad de México CP 11340, Mexico; mayra.s.p.flores@gmail.com (M.S.P.-F.); jtorresf@ipn.com (J.M.T.-F.); 4 Dirección Adjunta de Desarrollo Tecnológico, Vinculación e Innovación, Consejo Nacional de Humanidades Ciencias y Tecnologías, Ciudad de México CP 03940, Mexico; denys_8611@hotmail.com; 5 Laboratorio de Genética, Departamento de Zoología, Escuela Nacional de Ciencias Biológicas, Instituto Politécnico Nacional, Ciudad de México CP 11340, Mexico; leonavila60@yahoo.com.mx; 6 Laboratorio de Microbiología General, Departamento de Microbiología, Escuela Nacional de Ciencias Biológicas, Instituto Politécnico Nacional, Ciudad de México CP 11340, Mexico; abrilestela@hotmail.com

**Keywords:** NOD2, MAVS, DENV-2, macrophages, THP-1 macrophage-like cells, IFN-α, NOD2-siRNA

## Abstract

In pathogen recognition, the nucleotide-binding domain (NBD) and leucine rich repeat receptors (NLRs) have noteworthy functions in the activation of the innate immune response. These receptors respond to several viral infections, among them NOD2, a very dynamic NLR, whose role in dengue virus (DENV) infection remains unclear. This research aimed to determine the role of human NOD2 in THP-1 macrophage-like cells during DENV-2 infection. NOD2 levels in DENV-2 infected THP-1 macrophage-like cells was evaluated by RT-PCR and Western blot, and an increase was observed at both mRNA and protein levels. We observed using confocal microscopy and co-immunoprecipitation assays that NOD2 interacts with the effector protein MAVS (mitochondrial antiviral signaling protein), an adaptor protein promoting antiviral activity, this occurring mainly at 12 h into the infection. After silencing *NOD2*, we detected increased viral loads of DENV-2 and lower levels of IFN-α in supernatants from THP-1 macrophage-like cells with NOD2 knock-down and further infected with DENV-2, compared with mock-control or cells transfected with Scramble-siRNA. Thus, NOD2 is activated in response to DENV-2 in THP-1 macrophage-like cells and participates in IFN-α production, in addition to limiting virus replication at the examined time points.

## 1. Introduction

Dengue fever is one of the most prevalent arthropod-borne viral diseases affecting humans worldwide, with an estimated 390 million cases occurring annually in the tropical and subtropical regions of the world [1]. According to the World Health Organization (WHO), dengue significantly contributes to severe and incapacitating illness and mortality, particularly in Asian and Latin American countries. The virus comprises four antigenically related serotypes: DENV-1, DENV-2, DENV-3, and DENV-4, each exhibiting multiple genotypes [2]. The alternation between humans and mosquitos, coupled with the presence of multiple serotypes and genotypes, contributes to the complexity of understanding and combating this global health challenge.

DENV belongs to the Flavivirus genus of the *Flaviviridae* family. Mature DENV virions are enveloped and have a diameter of approximately 50 nm. The viral genome is composed of a positive-sense single-stranded RNA (ssRNA) molecule approximately 10.8 Kb long, enclosed in an icosahedral capsid, which is in turn surrounded by the viral envelope. The viral RNA has a single open reading frame (ORF) which encodes a polyprotein that is proteolytically cleaved by both viral and cellular proteases to produce three structural proteins (C, prM, and E) and seven non-structural proteins (NS1, NS2A, NS2B, NS3, NS4A, NS4B, and NS5) [3].

DENV is primarily transmitted to humans by the bite of *Aedes* spp mosquitoes, mainly *Aedes aegypti* and *Aedes albopictus*. Once inside its vertebrate host, DENV targets different cellular types, which include monocytes/macrophages, immature dendritic cells (iDCs), mature dendritic cells (mDCs), and reactive splenic lymphoid cells among others [4,5,6,7]. Within these cells, DENV triggers an innate immune response that performs as the first line of defense against infection. Afterward, the virus is recognized by pattern recognition receptors (PRRs), and a rapid response occurs, ending in the production of interferon type-I (IFNα/β) and inflammatory cytokines. Interferon (IFN) signaling alone induces hundreds of interferon-stimulated genes (ISGs) and modulates other branches of the immune response [8]. The collaboration between these effectors induces an antiviral state in both uninfected and virus-infected cells through several mechanisms already described [9].

In DENV-2-infected M1 macrophages which exhibit an inflammatory phenotype, NLRP3 inflammasome, a member of the NLR family, processes caspase-1, leading to the production of the active forms of IL-1β, IL-18, and pyroptosis [10]. In platelets, DENV modulates the release of IL-1β in micro-particles through mechanisms that might depend on mitochondrial activity [11]. However, the role of NOD2, another member of the NLR family and a described intracellular PRR in DENV infections, has not been entirely established.

NOD2 is a member of the NLR family, initially described as a cytosolic sensor for muramyl di-peptide (MDP) [12]. Currently, it is known that NOD2 is activated in the presence of genomes and metabolites from viruses, triggering and modulating immune signaling pathways related to nuclear factor kappa B (NF-κB), IFN regulatory factor (IRF) 3/7, and mitogen-activated protein kinase (MAPK), among others [13]. NOD2 activates IRF-3 transcription factor after interacting with mitochondrial antiviral signaling (MAVS) protein in cells infected by different negative sense ssRNA viruses [14]. Other reported interactions occur with receptor-interacting serine/threonine-protein kinase 2 (RIP2) [15,16] and 2′-5′-oligoadenylate synthetase 2 (OAS2) [17] enhancing antiviral signaling pathways.

MAVS actively participates in the induction of + antiviral responses through IFN type I, and its activation occurs via RIG-1 after detecting viral genomes in cytosol [18]. Distinct reports link anti-DENV activities mediated by different members of the OAS family, including OAS1 p42, OAS1 p46, and OAS3 p100. Nevertheless, various human OAS proteins appear to be differentially induced in various types of cells and are characterized by different subcellular locations and enzymatic parameters [19]. A recent report showed that increased levels of mRNA of OAS1, OAS2, and OAS3 are observed in U-937 macrophage-like cells infected with DENV-2 [20].

The aim of this research was to determine the role of NOD2 during DENV-2 infection in THP-1 macrophage-like cells in vitro. We focused on examining levels of NOD2 and possible interactions with the mentioned effector proteins; however, the interaction with MAVS stand out once observed through colocalization and co-immunoprecipitation. Because both antiviral activity and IFN type I-induction mediated by MAVS are well established, we analyzed IFN type I levels and virus titers following the silencing of NOD2. It is possible to conclude that in macrophage-like THP cells infected by DENV-2, NOD2 interacts with MAVS; then activation of IFN-α occurs, and these events impact viral titers. 

## 2. Materials and Methods

### 2.1. DENV-2 Strains, Culture, and Titration

The dengue virus employed in this study is a serotype 2 strain Yuc17438 (referred to as DENV-2) which was isolated from a patient in Yucatán, México. DENV-2 was cultivated on *Aedes albopictus* clone C6/36 confluent cell monolayers obtained from the American Type Culture Collection (ATCC, Manassas, VA, USA) and cultured at 28 °C in Leibovitz’s L-15 medium from Sigma-Aldrich (St. Louis, MO, USA), supplemented with L-glutamine, MEM non-essential amino acid solution, MEM vitamin solution, penicillin–streptomycin, 10% fetal bovine serum (FBS), and sodium pyruvate solution, all from Gibco (Waltham, MA, USA). Infections were carried out in Leibovitz’s L-15 medium supplemented with 2% FBS along with an uninfected control treated under equal conditions. Viral stock was prepared by infecting C6/36 cell monolayers seven days post-infection (dpi); the infected supernatants were harvested by centrifugation at 2000 rpm for 10 min and later were diluted to a final concentration of 8% with polyethylene glycol 8000 (Sigma-Aldrich) solution in PBS 1× (Gibco) and incubated overnight at 4 °C. The next day, the solution was centrifuged at 12,000 × rpm for 1 h. The pellet was re-suspended in 1/10 of the total volume in L-15 medium supplemented with 10% FBS, aliquoted, and frozen at −70 °C until use.

Viral stocks were titrated using a focus-forming assay with C6/36 cells. Briefly, ten-fold serial dilutions of viral stock were used to infect C6/36 cell monolayers with 200 µL of solution per well in 24-well plates. After 1 h of gentle agitation, each well was covered with 1 mL of overlaid medium consisting of 1% carboxymethyl cellulose (Sigma-Aldrich) in L-15 medium with 2% FBS. After 5 days, the overlaid medium was gently removed and the monolayers were fixed with 30% acetone in PBS 1× cold for 20 min at 4 °C; then the plaques were stained with a primary antibody of anti-DENV envelope (clone 4G2) from Merck-Millipore (Burlington, MA, USA) coupled with a secondary antibody and anti-mouse IgG-HRP from Invitrogen (Carlsbad, CA, USA). The foci were revealed using the substrate 3,3′-Diaminobenzidine (DAB) from Diagnostic Biosystems Inc. (Pleasanton, CA, USA). Viral titers were expressed as focus forming units per mL (FFU)/mL. An MOI of 10 was used for the following experiments.

### 2.2. THP-1 Cell Culture and Macrophage Differentiation

The THP-1 cell line (from human acute monocytic leukemia) was obtained from ATCC and cultured in RPMI-1640 medium from Gibco. RPMI-1640 medium was supplemented with sodium pyruvate solution, L-glutamine, penicillin–streptomycin 1×, MEM non-essential amino acid solution, and 10% FBS, all purchased from Gibco. The macrophage-like state was obtained by treating THP-1 cells over 3 days with 100 ng/mL of phorbol 12-myristate 13-acetate (PMA from Sigma-Aldrich). Afterward, adherent cells (THP-1 macrophage-like cells) were gently washed three times with PBS 1× from Gibco and rested with fresh RPMI-1640 medium for 5 days until use. Differentiation was verified, via the presence of CD14 marker in cells, using anti-human CD14 antibody conjugated to FITC (eBioscience, San Diego, CA, USA) in immunofluorescence assays (IFA). The antibody was incubated in paraformaldehyde (PFA)-fixed cells for 2 h at room temperature in the dark, and four washes were performed with PBS-Tween; then coverslips were mounted on slides using Vectashield (Vectorlabs, Burlingame, CA, USA). The slides were sealed and observed in an Axiovert200M microscope adapted to LSM 5 Pascal (Zeiss, Jena, Germany).

### 2.3. DENV-2 Infection in THP-1 Macrophage-like Cells

THP-1 cells were differentiated in THP-1 macrophage-like cells as described in Section 2.2 at 2 × 10^5^ cells/mL in 24-well plates from Corning (Corning, NY, USA). The infections were carried out using an MOI of 10, and the virus was diluted in 400 μL of RPMI medium. Virus was placed in contact with the cells, and its adsorption was allowed for 1 h with gentle stirring at 37 °C in 5% CO_2_. After incubation, the unabsorbed virus was removed by washing twice with 0.05% trypsin (Gibco) and once with PBS 1×. Finally, 1 mL of RPMI medium supplemented with 2.0% FBS was added and incubated at 37 °C in 5% CO_2_ until the infection period was completed. The supernatants were collected and centrifugated at 1500 rpm for 5 min and titrated by focus-forming assays as previously described. 

### 2.4. L18-MDP Treatment to Induce the Expression of NOD2 

NOD2 is a recognized general sensor of muramyl dipeptide (MDP) which responds to this interaction by activating NF-κB [21]. L18 muramyl dipeptide (L18-MDP 2 μg/mL) from Invivogen (San Diego, CA, USA) was used to transfect THP-1 macrophage-like cells as agonists to verify the positive expression of NOD2 at 3, 6, 12, and 24 h after transfection with Lipofectamine^TM^ 3000 Reagent (LP3000) from Invitrogen (Invitrogen, Carlsbad, CA, USA). Briefly, LP3000 was mixed with L18-MDP according to the manufacturer’s instructions and incubated at room temperature for 5 min. Later, the mix was added to the THP-1 monolayer for a final volume of 1 mL of Opti-MEM medium (Gibco) and incubated at 37 °C in 5% CO_2_ for the determined time; then cells were fixed and analyzed.

### 2.5. Poly I:C (PIC) Treatment to Induce MAVS Protein

MAVS is known to be activated by poly I:C (PIC 2 μg/mL), which was obtained from Sigma (Burlington, MA, USA) and used to transfect THP-1 macrophage-like cells as agonists to verify the positive expression of MAVS at 6, 12, and 24 h after transfection using Lipofectamine^TM^ 3000 Reagent (LP3000) from Invitrogen (Invitrogen, Carlsbad, CA, USA). LP3000 was mixed with PIC and incubated at room temperature for 5 min. Later, the mix was added to the THP-1 monolayer for a final volume of 1 mL of Opti-MEM medium (Gibco) and incubated at 37 °C in 5% CO_2_ for the determined time; then cells were fixed and analyzed.

### 2.6. Semi-Quantitative Reverse Transcription-Polymerase Chain Reaction (RT-PCR): mRNA Analysis

Total cellular RNA was isolated using TRIzol (Invitrogen) according to the manufacturer’s instructions. For reverse transcription (RT), the RNA was quantified in a NanodropTM from Thermo Sc. (Rockford, IL, USA), and then 1 μg of total RNA was added with 0.5 μg of oligo-dT (Thermo Sc.) and incubated for 10 min at 70 °C. The RT reaction included the following: 1× single strand buffer, 500 mM each deoxynucleotide triphosphate from Roche (Mannheim, Germany), and RevertAid reverse transcriptase (200 U) from Thermo Sc. Each RT reaction was incubated for 1 h at 42 °C to obtain cDNA. The PCR was performed using both cDNA and a commercial master mix with 2.0 mM MgCl_2_ from Ampliqon III (Odense M, Denmark). The human *GAPDH* gene was used as the endogenous control. The following primer sequences from Invitrogen were used for the RT-PCR:

*GAPDH*: forward 5′-GGTCATCCATGACAACTTTGG-3′, and reverse 5′-GTCATACCAGGAAATGAGCTTGAC-3′, 

*NOD2*: forward 5′-CAGCTGGACTACAACTCTGTGG-3′, and reverse 5′-GCAGAGTTCTTCTAGCATGACG-3′.

Amplified amplicons (*NOD2* 496 bp, and *GADPH* 414 pb) were analyzed on a 2% agarose gel and stained using ethidium bromide (Invitrogen). Gel images were acquired in a ChemiDoc-It™ transilluminator (UVP, Upland, CA, USA), and the integrated pixel density (PD) of each band was calculated using ImageJ (U. S. National Institutes of Health, Bethesda, MD, USA). The fold change was calculated by dividing the PD of NOD2 L18-MDP or DENV-2 by the PD of the mock specimen at the different time points. GADPH was used as a loading control.

### 2.7. Western Blot (WB)

For WB analysis, THP-1 macrophage-like cells differentiated in 6-well plates (2 × 10^6^ cells/well) were grouped as follows: untreated (mock), transfected (L18-MDP), and infected with DENV-2. After each treatment, the cells were lysed using RIPA buffer (50 mm Tris HCl pH 8, 150 mm NaCl, 1% NP-40, 0.5% sodium deoxycholic acid, and 0.1% SDS), which contained a protease inhibitor cocktail (Complete^TM^) from Roche Diagnostic GmbH (Roche, Mannheim, Germany). The protein extracts were forced through a 22-gauge needle 10 times and centrifuged for 10 min at 14,000 rpm at 4 °C. Total protein concentration was determined by PierceTM BCA protein assay kit from Thermo Fisher Scientific (Rockford, IL, USA) according to the manufacturer’s instructions. Forty µg of total protein was separated by 10% SDS–PAGE and transferred to a nitrocellulose membrane from Bio-Rad Laboratories (Berkeley, CA, USA). Membranes were blocked with 5% non-fat milk in PBS 1× for 1 h. After that, membranes were immunostained, and proteins were visualized using Luminata Forte from Merck-Millipore and the immunoreactive proteins were detected by exposure to Kodak Carestream film from Sigma-Aldrich. 

### 2.8. Confocal Microscopy

THP-1 cells (3 × 10^5^ cells/mL) were seeded on glass coverslips in a 24-well tissue culture plate (Corning, NY, USA). After the differentiation protocol, cells were categorized as untreated (mock), L18-MDP, or infected with DENV-2. Briefly, the viral stocks were adjusted to an MOI of 10 in a supplemented RPMI medium and incubated 1 h at 37 °C, in monolayers, followed by three washes with PBS 1× (Gibco). After the indicated times, cells were fixed with PFA 4% for 15 min at room temperature. Cells were permeabilized with 0.1% Triton X-100 (Sigma-Aldrich) in 1 × PBS for 15 min and blocked with 5% bovine serum albumin (Sigma-Aldrich) for 1 h at room temperature. Afterward, the following primary antibodies were added: anti-NOD2, or anti-MAVS, from Genetex Inc. (Irvine, CA, USA); each antibody was incubated for 2 h and washed three times with PBS 1×. Then, secondary antibodies were added as follows: an anti-mouse IgG coupled with Dylight-488 antibody from Invitrogen was used for NOD2, and for MAVS, an anti-rabbit IgG coupled with APC antibody from Santa Cruz Biotechnology Inc. was incubated for 30 min in the dark at room temperature. Finally, the nuclei were labeled with DAPI obtained from Sigma-Aldrich for 15 min, and the slides were mounted with Vectashield from Vector Laboratories (Burlingame, CA, USA). The images were captured using a confocal laser scanning system (LSM5 Pascal) attached to a confocal microscope (Axiovert 200 M) from Zeiss (Jena, Germany), and 100 cells were captured for each condition. Cell culture supernatants were collected and frozen at −70 °C for cytokine measurement or viral titration.

### 2.9. Co-Immunoprecipitation Assays (Co-IP)

For Co-IP assay, THP-1 macrophage-like cells were differentiated in T-25 cell culture flasks (5 × 10^6^ cells/flask); they were untreated (mock), transfected (PIC), or infected with DENV-2. After the incubation period of each treatment was completed, the cells were lysed using RIPA buffer containing a cocktail protease inhibitor (previously mentioned). Total protein lysates were quantified as previous described, and 150 µg of total protein was incubated with anti-NOD2 antibody (2 µg/reaction) overnight at 4 °C with constant rocking. Afterward, 15 μL of protein A agarose beads from Thermo Sc. was added to each mixture and incubated with gently mixing while the tube was inverted constantly at 4 °C for 3 h. Subsequently, precipitates were washed eight times with PBS 1×-protease inhibitors. Later, the pellets were re-suspended, and MAVS presence was evaluated via immunoblot assay. Immunoblots were probed using anti-MAVS (1 µg/mL) coupled with an anti-rabbit IgG-HRP conjugated antibody and visualized using enhanced chemiluminescence using Luminata Forte (Merck-Millipore).

### 2.10. Small Interfering RNA (siRNA) Assays

A total of 2.5 × 10^6^ THP-1 macrophage-like cells in 6-well plates were transfected using a mix of two siRNAs, *Card4* (25 pmol) and *Card6* (25 pmol), against human *NOD2* (referred in the text as NOD2-siRNAs, catalog number 1027415) from Qiagen (Hilden, Germany). As a negative control, AllStars negative control siRNAs (referred to in the text as Scramble-siRNA 50 pmol) (catalog number 1027281, from Qiagen) were transfected using LP3000 (Invitrogen) according to the manufacturer’s protocol for 12 h. After siRNA transfection, THP-1 macrophage-like cells were analyzed in three groups: mock (untreated), DENV-2 infected, and transfected with PIC (positive control). Cell viability was measured using MTT (Sigma-Aldrich); briefly, 15 µg of MTT reagent (500 µg/mL) was added to each well and incubated for 3 h at 37 °C; then cells were lysed with 100 µL of DMSO (Sigma-Aldrich) and rocked until the complete solubilization of purple formazan crystal, and absorbance was measured at 600 nm in a microplate reader from Thermo Sc. Absorbance was used to calculate the percentage of cell viability as follows: (Atreatment—Ablank)/(Acontrol—Ablank) × 100%. Additionally, the efficiency of NOD2 knocked down was evaluated by Western blot as described elsewhere. 

### 2.11. IFN-α Quantification

The concentration of IFN-α was measured in THP-1 macrophage-like cell culture supernatants by enzyme-linked immunosorbent assay (ELISA), using a human IFN-α kit from eBioscience (San Diego, CA, USA); the measurements were taken according to the manufacturer’s instructions.

### 2.12. Statistical Analysis

The statistical testing was performed using GraphPrism^®^ version 8.0.1 (GraphPad Software Inc., San Diego, CA, USA). For comparison between the three groups, one-way ANOVA was used to determine differences. For comparison between two groups of continuous variables, they were analyzed with Student’s *t*-test in the case of parametric distributions. The specific statistical analysis performed is indicated for each figure. Descriptions of experimental replicates are also described in the figure legends. The results are shown as the mean ± standard error of the mean (SEM). *p* values ≤ 0.05 were considered statistically significant.

## 3. Results

### 3.1. Infection with DENV-2 Upregulates NOD2 Expression in THP-1 Macrophage-like Cells

**Figure 1:**
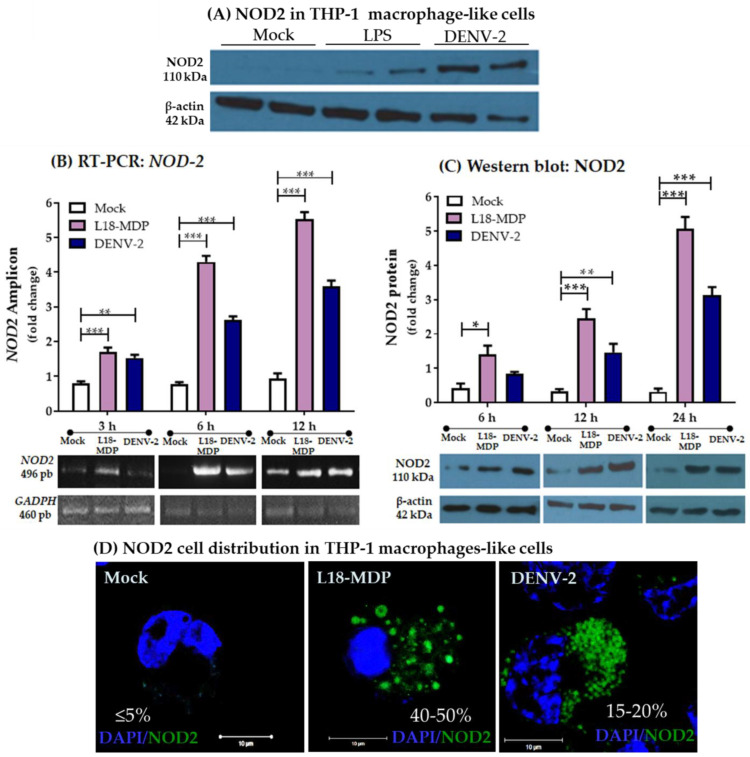
Detection of NOD2 in THP-1 macrophage-like cells stimulated with L18-MDP or infected with DENV-2. (**A**). NOD2 protein was detected as early as 3 hpi in cells infected by DENV-2 (MOI of 10) and in the positive control with LPS (1 µg/mL). (**B**,**C**). NOD2 expression was assessed at different time points in THP-1 macrophage-like cells under different treatments: mock (white bars), positive control transfected with L18-MDP (pink bars) or DENV-2-infected at an MOI of 10 (dark blue bars). (**B**). *NOD2* mRNA was measured after 3, 6, and 12 h using end point RT-PCR using specific primers for human *NOD2* and *GAPDH*. Bar graph indicates fold change calculated by the ratio *NOD2*/*GAPDH.* (**C**). NOD2 protein expression was evaluated by Western blotting of the whole-cell lysates using an anti-NOD2 antibody and an anti-β-actin antibody as a loading control. Bar graph indicates the fold change calculated by the ratio NOD2/β-actin. All experiments were performed three times. Statistical differences were assessed with one-way ANOVA, where *** *p* < 0.001, ** *p* < 0.01, and * *p* < 0.05. (**D**) Confocal micrograph of THP-1 macrophage-like cells that have undergone the treatments indicated in the images for 12 h, followed by immunostaining for NOD2 (green), with the nuclei contrasted (blue). Images show the percentage of NOD2-positive cells which were found after counting 100 cells in each condition. Pictures were cropped to improve presentation, the digital magnification is 2100×, and the scale bar indicates 10 µM.

NOD expression was verified in THP-1 macrophage-like cells differentiated from macrophages in three experimental groups: mock, positive control treated with LPS (1 µg/mL), and DENV-2-infected cells in two independent experiments examined as early as 3 h after each treatment, as observed in Figure 1A (Supplementary Figure S1A). Different inducers of NOD2 expression have been described; we initially tested LPS, L18-MDP, and PIC to analyze the changes in NOD2 expression in THP-1 macrophage-like cells. However, for subsequent experiments, only L18-MDP and PIC were used as agonists (positive control) supported by the initial analysis. To characterize the kinetics of NOD2 at the mRNA and protein levels in THP-1 macrophage-like cells, specific primers were used to obtain an amplicon for *NOD2* mRNA, and Western blot assays were used to examine NOD2 protein at three different time points; we used a NOD2-specific inducer (L18-MDP), and THP-1 macrophage-like cells infected by DENV-2 and mock control cells as shown in Figure 1B,C. NOD2-related amplicon in DENV-2-infected THP-1 macrophage-like cells showed an increased expression starting at 3 hpi (1.5 ± 0.3-fold), followed by 6 hpi (2.7 ± 0.2-fold) and 12 hpi (3.7 ± 0.2-fold) compared with mock infected cells. In the same comparison, treatment with L18-MDP, an agonist of NOD2 (positive control), resulted in the highest increase of NOD2 expression, starting at 3 h (1.6 ± 0.3-fold), continuing at 6 h (4.3 ± 0.4-fold) and 12 h (5.6 ± 0.3-fold) in comparison with mock infected cells (Figure 1B and Supplementary Figure S1B). Similarly, Western blot was used to assess protein levels of NOD2 in THP-1 macrophage-like cells infected with DENV-2, which were higher, as noticed at 12 hpi (1.5 ± 0.3-fold) and 24 hpi (3.1 ± 0.2-fold) in comparison with the mock-infected control. Meanwhile, THP-1 macrophage-like cells transfected with L18-MDP (positive control) showed higher levels of NOD2 protein at each time point, with 1.4 ± 0.4-fold at 6 h, 2.5 ± 0.4-fold at 12 h, and 5 ± 0.5-fold at 24 h (Figure 1C).

We evaluated the cellular localization of NOD2 in THP-1 macrophage-like cells by confocal microscopy, in a mock control; the expression of this molecule occurred at a low level (Figure 1D). In THP-1 macrophage-like cells infected with DENV-2, NOD2 was localized in cytosol in a characteristic punctate pattern that accounted for 15 to 20% of cells (*n* = 100 cells examined in 3 independent experiments) at 12 hpi. In contrast, THP-1 macrophage-like cells transfected with the synthetic L18-MDP exhibited a vesicle-like pattern of distribution for this protein in nearly to 40 to 50% (*n* = 100 cells examined in 3 independent experiments) of the examined cells at 12 h. Although macrophages derived from the THP-1 cell line are reportedly permissive for DENV-2 infection in vitro [22], as control for DENV-2 infection, we evaluated the expression of viral NS3 protein (red), which was detected at 12 hpi. In THP-1 macrophage-like cells infected with DENV-2, NOD2 was found in the cytosol, colocalizing with NS3; the infected cells positive for both NOD2 and NS3 corresponded to approximately 18% of cells at this early time (Supplementary Figure S2). Our findings suggest that viral infection is needed to upregulate NOD2 in these cells. 

### 3.2. Detection of MAVS Effector and Interaction with NOD2 in THP-1 Macrophage-like Cells

**Figure 2:**
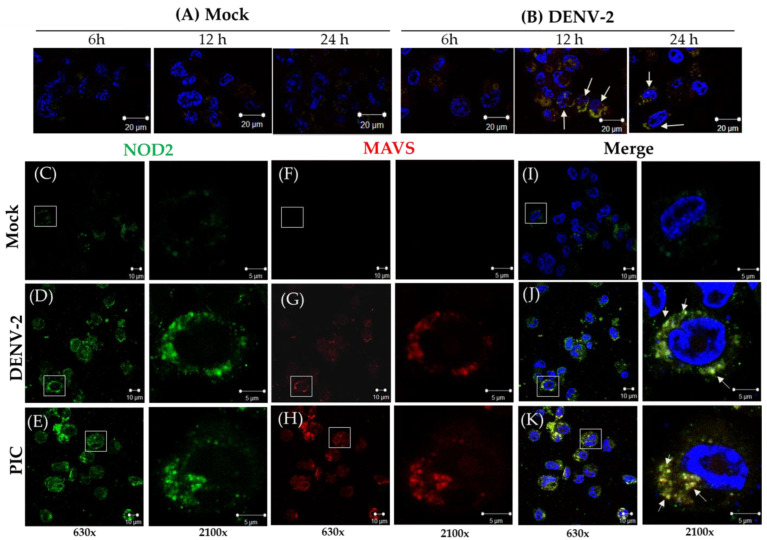
DENV-2 induces NOD2–MAVS colocalization in THP-1 macrophage-like cells. (**A**,**B**) Representative confocal micrographs shown in (**A**) THP-1 macrophage-like cells with a mock treatment and (**B**) DENV-2-infected cells at 6, 12, and 24 h. Fluorescence images show the merge fields of NOD2 (green), MAVS (red), and nuclei (blue). Arrows indicate the cells where colocalization was observed. The scale bars indicate 20 µm, magnification 630×. (**C**–**K**) Representative confocal micrographs of the colocalization (**C**–**E**) of NOD2 (green) and (**F**–**H**) MAVS (red) in THP-1 macrophage-like cells in the following groups: mock, DENV-2-infected, and positive control (PIC) at 12 h. (**I**–**K**) On the far right, the images of NOD2 and MAVS are merged, with a nuclear contrast (blue). Individual cells displayed in the square were selected for observation in greater detail. A digital magnification of 630× is illustrated on the left and one of 2100× on the right side of each column. The arrows indicate points of NOD2–MAVS colocalization. Each condition was repeated three times.

**Figure 3:**
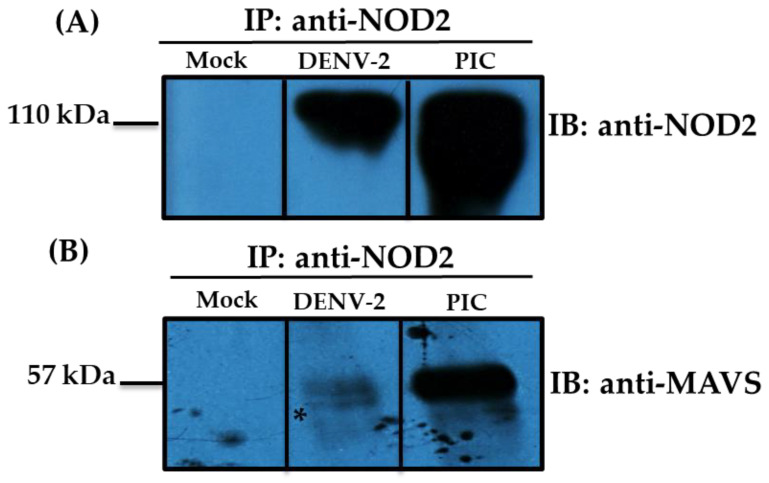
DENV-2 induces the NOD2–MAVS interaction in THP-1 macrophage-like cells after 12 hpi. NOD2 interaction with MAVS was evaluated via immunoprecipitation (IP) and Co-IP assays in whole protein cell lysates of THP-1 macrophage-like cells. THP-1 macrophage-like cells were grouped as follows: mock, DENV-2-infected, and the positive control (cells transfected with PIC). (**A**) Western blot image of the IP with an anti-NOD2 antibody, and IB with the same antibody shows an increase in NOD2 production in the positive control (PIC), and in cells infected with DENV-2 at 12 h. (**B**). Western blot image of the Co-IP shows the interaction of NOD2–MAVS in the positive control (PIC) and in cells infected with DENV-2 (asterisk) at 12 hpi. Proteins were IP with an anti-NOD2 antibody and IB with an anti-MAVS antibody. The images show one representative experiment. Uncropped membranes are presented in Supplementary Figure S3A).

We assessed the levels of MAVS effector in mock and DENV-2-infected THP-1 macrophage-like cells at three different time points (6, 12, and 24 h) by confocal microscopy (Figure 2A,B). The best signal for MAVS was observed at 12 h (Figure 2B,J); thus, for further analysis to colocalize NOD2 and MAVS, we used that time point. MAVS responds to the stimuli of the agonist PIC. In the mock THP-1 macrophage-like cells, colocalization of NOD2–MAVS was not observed (Figure 2C,F,I); meanwhile for DENV-2 infected cells, colocalization was detected in the 35–50% of the NOD2-positive cells (Figure 2D,G,J); for the THP-1 macrophage-like cells transfected with PIC (positive control), colocalization was observed in 50 to 60% of the NOD2-positive cells (Figure 2E,H,K). Increased expression of MAVS correlating with the expression of NOD2 was verified in the cytosol of DENV-2 infected cells (Figure 2D,G,J). Subsequently, Co-IP assays were carried out to verify the protein interactions of MAVS with NOD2 protein (Figure 3). MAVS protein was recovered when using an anti-NOD2 antibody to coimmunoprecipitate and anti-MAVS antibody for the immunoblot. MAVS protein was recovered both in lysates of THP-1 macrophage-like cells transfected with PIC (positive control) and in cells infected by DENV-2 at 12 h of the stimulus, but not in mock cells (Figure 3A and Supplementary Figure S3A). Our data confirmed the NOD2–MAVS interaction in the THP-1 macrophage-like cells infected with DENV-2. To corroborate the presence of NOD2 in this assay, NOD2 was immunoprecipitated employing an anti-NOD2, and the immunoblots were performed with the same antibody (Figure 3B and Supplementary Figure S3B). 

### 3.3. Downregulation of NOD2 Reduces the Production of IFN-α in Macrophage-like Cells Infected by DENV-2

**Figure 4:**
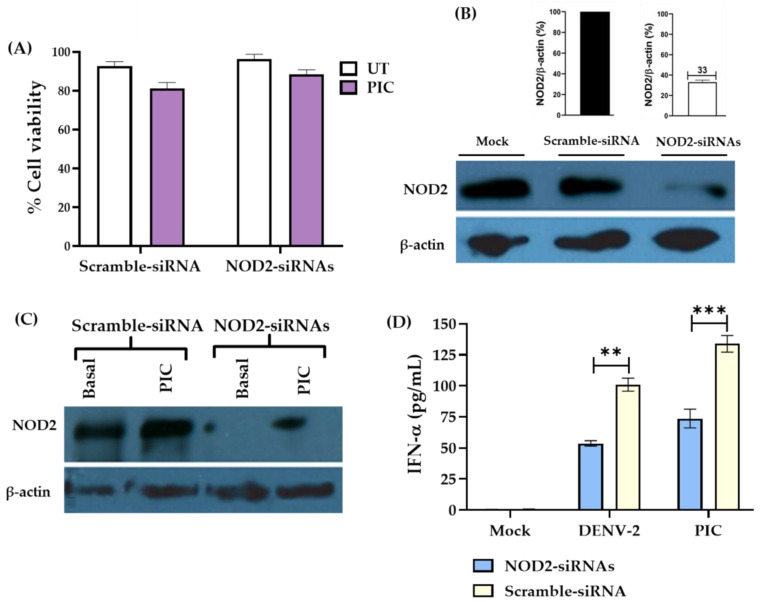
Analysis of THP-1 macrophage-like cells knocked down in *NOD2* by specific NOD2-siRNAs shows a diminished production of IFN-α in THP-1 macrophage-like cells infected with DENV-2. (**A**) Quantification of cellular viability in THP-1 macrophage-like cells were performed via MTT assay. Bar graphs represent the mean ± SEM of the percentage of cell viability in THP-1 macrophage-like cells transfected with a Scramble-siRNA or NOD2-siRNAs and after 24 h of stimulation with the agonist PIC or untreated. A paired *t*-test was performed for statistical analysis to compare UT vs. PIC in each group; no differences were observed. N = 3. (**B**) Western blot of the silencing assay shows that NOD2 was downregulated in THP-1 macrophage-like cells transfected with specific NOD2-siRNAs. Bar graphs shown the percentage of the ratio NOD2/β-actin. (**C**) Western blot of THP-1 macrophage-like cells transfected with specific NOD2-siRNAs and stimulated with the agonist PIC produce lower levels of NOD2 protein than the cells transfected with the Scramble-siRNA and stimulated with the agonist PIC. (**D**) THP-1 macrophage-like cells were silenced with a mix of specific human NOD2-siRNAs or with a Scramble-siRNA (negative control); earlier, the cells were stimulated with PIC and infected with DENV-2; after 24 h, the cell supernatants were collected, and IFN-α levels were measured by ELISA. Bar graphs shows the IFN-α levels (pg/mL) mean ± SEM of three independent experiments. Statistical differences were evaluated by one-way ANOVA, where *** *p* < 0.001, and ** *p* < 0.01.

Later, the effect of downregulating NOD2 in THP-1 macrophage-like cells and the response in the tested groups was evaluated. We employed gene silencing with specific human NOD2-siRNAs. First, we addressed by MTT assays the percentage of cell viability of THP-1 macrophage-like cells subjected to transfections with siRNAs and with the agonist (PIC)**.** Our analysis showed a high percentage of cell viability (80–88%), in cells exposed to double transfections (Figure 4A).

To assure NOD2 expression was reduced globally in around 67% of transfected with specific NOD2-siRNAs cells compared with those transfected with a Scramble-siRNA or mock control; these results were confirmed by Western blot (Figure 4B, and Supplementary Figure S4A). The expression of NOD2 was not restored to normal regardless of using PIC agonist in silenced cells (Figure 4C and Supplementary Figure S4B). 

Our previous results where the interaction of NOD2–MAVS was identified in THP-1 macrophage-like cells infected with DENV-2 (Figure 2J and Figure 3A), made us ask if the production of IFN-α would be affected in these cells. Data showed that cells transfected with a Scramble-siRNA and stimulated with the IFN-α agonist PIC showed levels of around 136 ± 3 pg/mL; however, cells transfected with specific NOD2-siRNAs and stimulated with PIC showed lower levels of IFN-α, around 87 ± 4 pg/mL (Figure 4D). Similarly, the cells transfected with a Scramble-siRNA and infected with DENV-2 showed levels around 116 ± 3 pg/mL, whereas cells transfected with NOD2-siRNAs and infected with DENV-2 had lower IFN-α production, around 63 ± 4 pg/mL. IFN-α was not detected in untreated cells (UT) (Figure 4C). 

### 3.4. NOD2 Inhibition Increased Viral Loads in THP-1 Macrophage-like Cells

Since our data suggest that NOD2 may be important in the induction of antiviral response [14], we asked whether decreasing NOD2 expression in THP-1 macrophage-like cells infected with DENV-2 would have any effect on viral load, something plausible based on our previous data, in which the THP-1 macrophage-like cells downregulated NOD2 expression and those treated with the agonist PIC or infected with DENV-2 secreted lower levels of IFN-α than cells transfected with a Scramble-siRNA or in the mock cells.

**Figure 5:**
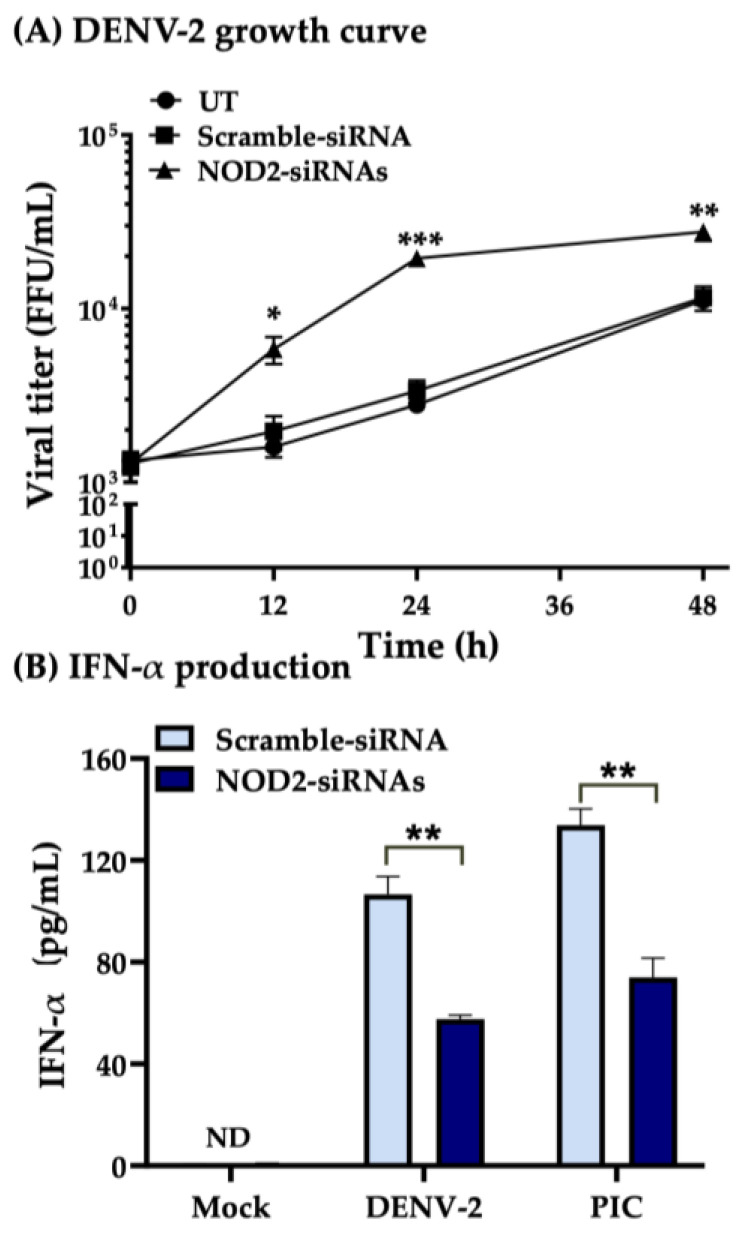
Silencing *NOD*2 in THP-1 macrophage-like cells infected with DENV-2 increases viral titers and affects IFNα levels. (**A**) DENV-2 growth curve. THP-1 macrophage-like cells were untreated (UT) or transfected with a Scramble-siRNA or with an NOD2-siRNAs after all groups were infected with DENV-2 for 12, 24, and 48 h. The graph shows the viral titers of the three experimental groups expressed as FFU/mL mean ± SEM of three independent experiments at 12, 24, and 48 h. The DENV-2 growth curve shows comparisons between the viral loads in THP-1 macrophage-like cells treated with Scramble-siRNA or NOD2-siRNAs vs. untreated cells at each time point. The statistical differences to each time point of the curve were analyzed with one-way ANOVA. *** *p* < 0.001, ** *p* < 0.01, and * *p* < 0.05. The viral progeny was quantified by focus-forming assay in C6/36 cells. (**B**) IFN-α levels were quantified in supernatants of THP-1 macrophage-like cells at 24 h post-infection with DENV-2 in mock cells or transfected with Scramble-siRNA or NOD2-siRNAs. Bar graph shows the IFN-α levels (pg/mL) mean ± SEM of three independent experiments. Statistical differences were evaluated by paired *t*-test, where ** *p* < 0.01.

The THP-1 macrophage-like cells which were knocked down in NOD2 expression with specific NOD2-siRNAs produced higher viral loads (1.8 ± 0.3 × 10^4^ FFU/mL, more than 4-fold) than those transfected with a Scramble-siRNA (4.6 ± 0.5 × 10^3^ FFU/mL) at 24 hpi (Supplementary Figure S5A). To validate our previous results, we analyzed the viral progeny in THP-1 macrophage-like cells knocked down in NOD2 at different time points. The viral progeny followed a growth curve at different time points (12, 24, and 48 hpi). Our data showed an increased viral load in THP-1 macrophage-like cells treated with the NOD2-siRNA, which was time-dependent beginning at 12 hpi; however, higher differences were found at 24 hpi (1.9 ± 0.18 × 10^4^ FFU/mL) and 48 hpi (2.7 ± 0.31 × 10^4^ FFU/mL) when compared with untreated THP-1 macrophage-like cells (2.9 ± 0.5 × 10^3^ FFU/mL at 24 hpi, and 1.1 ± 0.18 × 10^4^ FFU/mL at 48 hpi) or transfected with a Scramble-siRNA (3.3 ± 0.3 × 10^3^ FFU/mL at 24 hpi, and 1.1 ± 0.31 × 10^4^ FFU/mL at 48 hpi) (Figure 5A). These results confirm the role of the NOD2–MAVS interaction in the partial induction of an antiviral state mediated by IFN-α in THP-1 macrophage-like cells infected with DENV-2 as shows Figure 5B.

## 4. Discussion

PRRs are the first line of recognition for defense against pathogens in cells. During viral infections, the activation and potentiation between two kinds of PRRs, the TLR and RLR families, have been reported on. However, in the NLR family, there are members of great importance for inflammatory functions such as NLRP3 [10,11] or NLRX1, which regulatory functions on RIG-I/MAVS pathway and are important in viral infection [23], and NOD2 [14,15,16,17], a less studied effector in viral infections.

In cells such as monocytes or macrophages and dendritic cells, there is an immediate response mediated by TLR3, TLR7, TLR8, RIG-I, and NLRP3, which elicit a rapid and coordinated antiviral response in the early stages of DENV-2 infection [10,24,25,26]. Nevertheless, other intracellular sensors located in cytosol can be activated and participate in the establishment of this response. Among them, NOD2 is a very dynamic molecule than interacts with several effectors that intervene in either proinflammatory or antiviral immune response. Our results experimentally proved that, according to RT-PCR and Western blot testing, NOD2 induction is augmented in THP-1 macrophage-like cells in response to DENV-2. We observed singular patterns of subcellular distribution for the expression of NOD2 in THP-1 macrophage-like cells after the treatment with the agonist L18-MDP or DENV-2, suggesting that NOD2 may be present in defined vesicles such endosomes, as has been reported elsewhere [27]. However, to identify the nature of those vesicles, further studies will be needed. 

An interesting finding is that THP-1 cells expressing the higher levels of NOD2 are also those positive for DENV-2 infection, as confirmed by the presence of viral NS3. These data suggest that active DENV-2 replication is necessary to elicit the upregulation of NOD2. So far, this molecule has been associated only with significant homeostatic regulation in intestinal epithelial cells [28]. However, the involvement of the NOD2 molecule in processes important for proinflammatory and antiviral immune response has been suggested [29]. The rationale led us to investigate the role of NOD2 during DENV-2-infection in this experimental model, which represents one of the main targets and more significant cells for the immune response to dengue: macrophages. According to previous reports, during infection with several RNA viruses, NOD2 is activated and leads to the activation of a protective innate immune response mediated by interactions with adaptors including RIP2, MAVS, OAS2, CARD9, etc., resulting in activities against the pathogens [30]. 

Protein interactions with numerous effectors for NOD2 might be significant for the course and final immune response [31]. Colocalization of NOD2 was examined by confocal microscopy for molecules with antiviral activity, such as RIP2, MAVS, and OAS2; of these, NOD2–MAVS was the main interaction observed. To further investigate these discoveries, we analyzed the NOD2–MAVS interaction by Co-IP, and data showed that NOD2 can interact with MAVS in DENV-2 infected cells or in the cells stimulated by the agonist PIC at different times post-stimulus. The overall mechanism needs deeper study.

NOD2 downregulation by specific siRNAs correlates with a decreased secretion of IFN-α in cells infected with DENV-2; it is important to point out that this trait must be examined in the entire organism, where most complex cytokine interactions play important roles. 

Next, we evaluated whether NOD2 misfunction in THP-1 macrophage-like cells infected with DENV-2 had an impact on viral loads. Higher viral titers in experimental groups of THP-1 cells with a decreased expression at different times post-infection were observed. Taken together with the findings herein presented, our data confirmed a new role for NOD2–MAVS interaction during DENV-2 infection in THP-1 macrophage-like cells, which consisted of limiting the production of new viral progeny. We found this a very significant finding that indicates that NOD2 either potentiates or initiates the antiviral response to limit virus progeny. This trait has been confirmed in other viral infections, such as foot-and-mouth disease, to counteract NOD2 to antagonize antiviral activity [14,32,33,34].

## 5. Conclusions

In conclusion, the receptor NOD2 is upregulated and activated by an active viral replication in macrophages derived from the THP-1 cell line at early stages of the infection with DENV-2. The activation of NOD2 during the infection leads to interaction with MAVS and elicits the secretion of IFN-α. Importantly, NOD2 participates in limiting the production of new DENV-2 viral particles in the cell.

## Data Availability

All the data generated during the current study are included in the manuscript.

## Supplementary Materials

The following supporting information can be downloaded at: https://www.mdpi.com/article/10.3390/pathogens13040306/s1, Figure S1. (A, and B) Western blot analysis of whole cell lysates of THP-1 macrophage-like cells. Figure S2. NS3 and NOD2 expression in THP-1 macrophage-like cells infected with DENV-2. Figure S3. Coinmmunoprecipitation (Co-IP) analysis of the interaction between NOD2 and MAVS in THP-1 macrophages-macrophage-like cells. Figure S4. DENV-2 induces the interaction NOD2–MAVS in THP-1 macrophage-like cells. Figure S5. DENV-2 titers in THP-1 macrophage-like cells at 24 hpi.
