# Supplementary material for: NOD2 Responds to Dengue Virus Type 2 Infection in Macrophage-like Cells Interacting with MAVS Adaptor and Affecting IFN-α Production and Virus Titers
Source: Pathogens. 2024 Apr 10;13(4):306. doi: 10.3390/pathogens13040306 (PMC11054756; doi:10.3390/pathogens13040306)
Supplement: Supplementary file 1 [file pathogens-13-00306-s001.zip › pathogens-2905916-supplementary.pdf]

Supplementary Figure S1

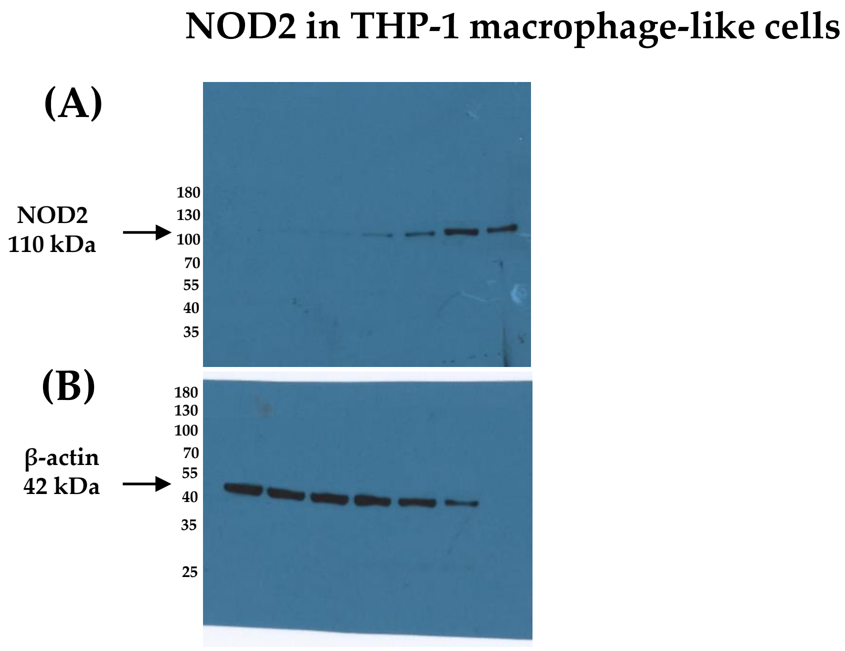

Supplementary Figure S1. (A, and B) Western blot analysis of whole cell lysates of THP-1 macrophages-like cells**.** Uncropped full-length pictures of Western blot membranes presented in **Figure 1A**. The membranes were homogenized in brightness and contrast to improve their presentation. The arrows indicate the band of interest.

Supplementary Figure S2

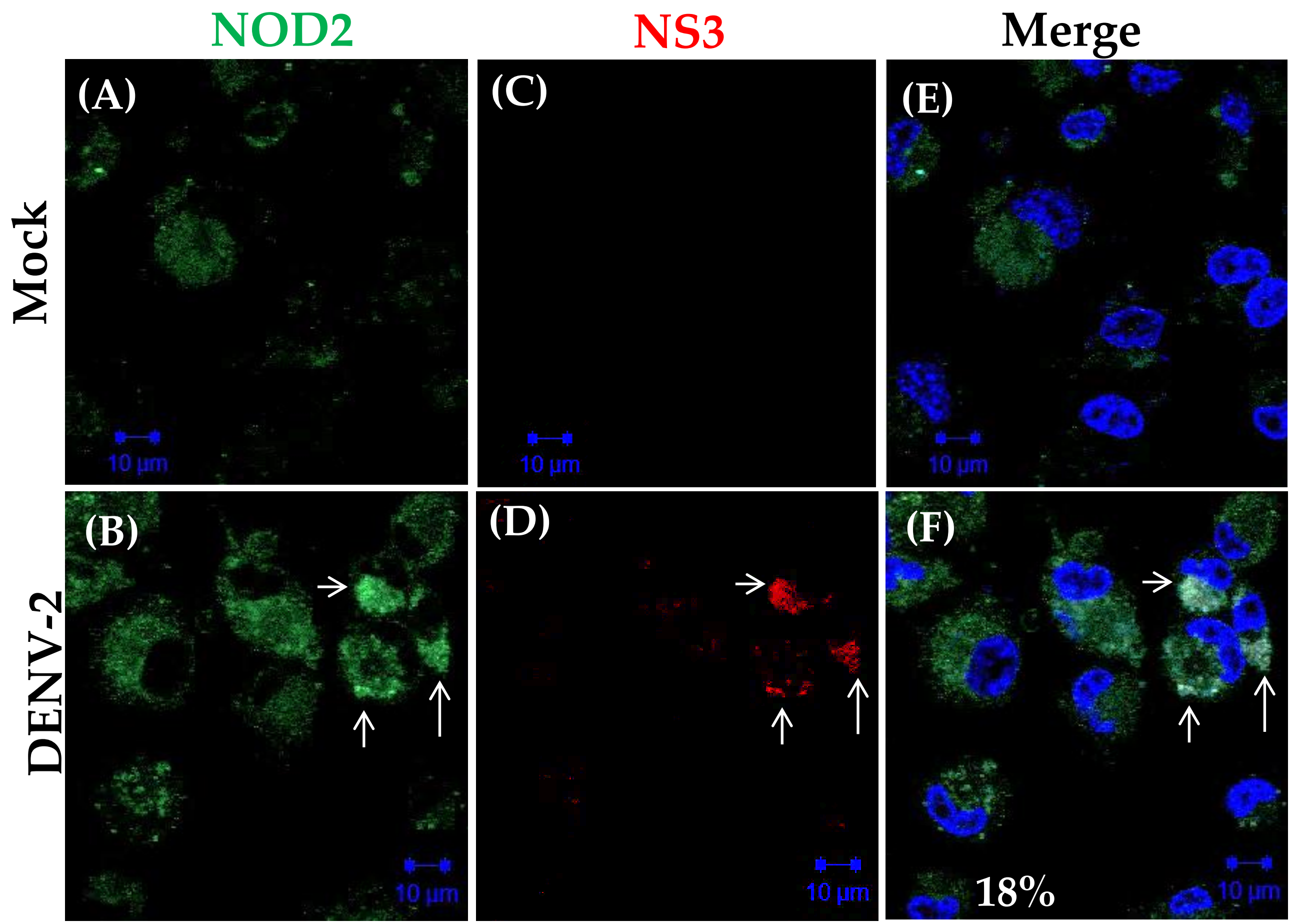

Supplementary Figure S2. NS3 and NOD2 expression in THP-1 macrophages-like cells infected with DENV-2. NOD2 and viral NS3 were evaluated at 12 h by confocal microscopy. Representative images of NOD2 (green), viral NS3 (red), and nuclei (blue) in THP-1 macrophages-like cells un-infected (Mock) **(A, C, and E)** or DENV-2-infected cells **(B, D, and F).** Arrows are indicating a positive cells to NOD2 and viral NS3 which correspond to approximately 18 percent of the cells. The scale bars indicate 10 µm with a magnification of 630×. 100 cells were captured in random microscope fields of three independent experiments.

Supplementary Figure S3

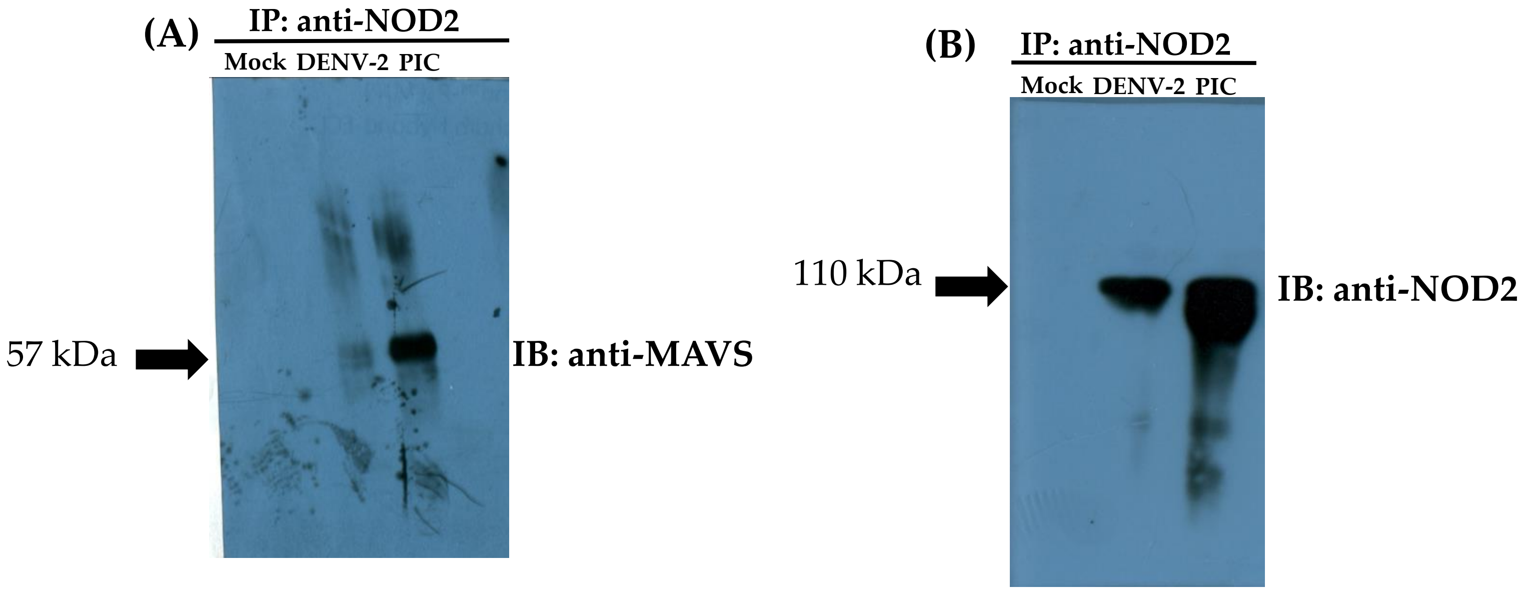

Supplementary Figure S3. Coinmmunoprecipitation (Co-IP) analysis of the interaction between NOD2 and MAVS in THP-1 macrophages-macrophages like cells. (A) Co-IP assay were performed using an anti-NOD2 antibody followed by a Western blot analysis using an anti-MAVS antibody. (**B**) IP assay using an anti-NOD2 antibody followed by a Western blot analysis using the same antibody. Uncropped full-length pictures of Western blot membranes presented in **Figure 3A and B**. The membranes were homogenized in brightness and contrast to improve their presentation. The arrows indicate the band of interest.

Supplementary Figure S4

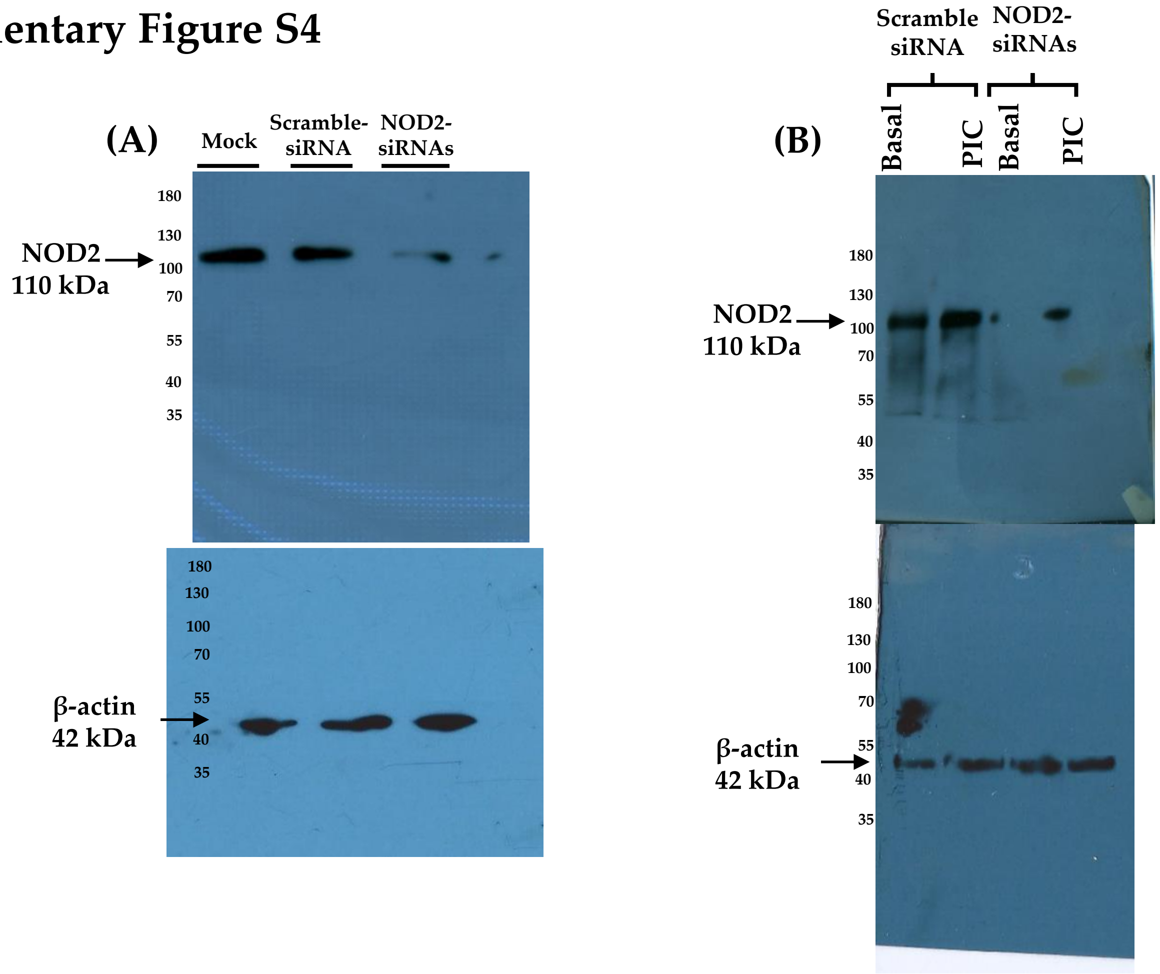

Supplementary Figure S4. DENV-2 induces the interaction NOD2-MAVS in THP-1 macrophages-like cells. (A) Western blot analysis of whole cell lysates of THP-1 macrophages-like cells transfected with Scramble-siRNAs or NOD2-siRNAs and the Mock cells at 24 h. (**B**) Analysis of the NOD2 in THP-1 macrophages like-cells knocked down with specific NOD2-siRNAs and stimulated with the agonist PIC (positive control). Uncropped full-length pictures of Western blot membranes presented in **Figure 4A, and B**. The membranes were homogenized in brightness and contrast to improve their presentation. The arrows indicate the band of interest.

Supplementary Figure S5

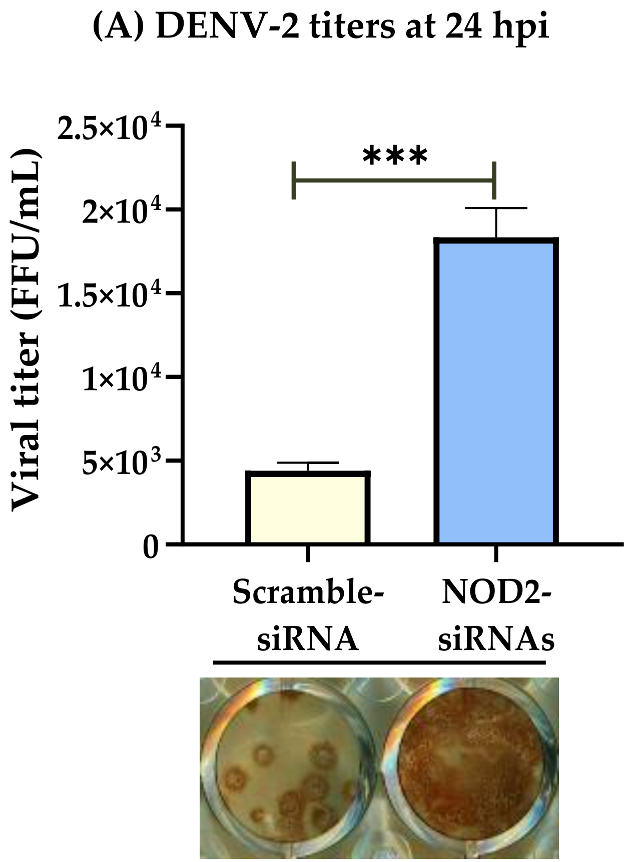

Supplementary Figure S5. DENV-2 titers in THP-1 macrophages-like cells at 24 hpi. (A) Bar graphs represent the mean ± SEM of the percentage of FFU/mL in the supernatants of THP-1 macrophages-like cells transfected with a Scramble-siRNA or NOD2-siRNAs, and later of 24 h of infection with DENV-2. A paired T-test was performed for statistical of three independent experiments. *** $p < 0.001$.
